# Sensory nerve conduction studies in infants, children and teenagers – An update

**DOI:** 10.1016/j.cnp.2024.01.001

**Published:** 2024-01-08

**Authors:** Tom Frenzel, Anne-Katrin Baum, Hardy Krause, Christoph Arens, Aiden Haghikia, Imke Galazky

**Affiliations:** a Dept. of Neurology, Otto-von-Guericke University, Magdeburg, Germany; b Dept. of Pediatric Surgery, Otto-von-Guericke University, Magdeburg, Germany; c Dept. of Otorhinolaryngology, Otto-von-Guericke University, Magdeburg, Germany; d Dept. of Otorhinolaryngology, Justus-Liebig University, Giessen, Germany

**Keywords:** Sensory nerve conduction study, Children, Analgosedation, Reference values, Acceleration

## Abstract

**Objective:**

Nerve conduction studies (NCS) in children remain technically challenging and depend on the cooperation of the child. Motor NCS are not compromised by analgosedation but data for sensory NCS are lacking. Here, we ask whether sensory NCS is influenced by analgosedation. We also compare the present data with NCS studies from the 1990s regarding anthropometric acceleration of the contemporary paediatric population.

**Methods:**

Sensory NCS of the median nerve and sural nerve were performed in 182 healthy subjects aged 1 to 18 years during general anaesthesia and in 47 of them without analgosedation.

**Results:**

Sensory NCS was not influenced by midazolam or propofol. The sensory nerve action potential (SNAP) amplitude and the nerve conduction velocity (NCV) of the sural nerve as well as the SNAP of the median nerve show no significant age dependence in age range 1–18 years. The sensory NCV of the median nerve increased age-dependent.

**Conclusions:**

In clinical practice, analgosedation can be used for diagnostic NCS. Sensory NCS data show no relevant secular trend over the last 30 years. Differences due to technical inconsistency predominate.

**Significance:**

Analgosedation can improve diagnostic quality of sensory NCS in children. Additionally, we provide sensory NCS values from a large pediatric cohort.

## Highlights

• Diagnostic sedation and anxiolysis with midazolam or propofol can be considered for sensory NCS.
• We here provide reference values of sensory NCS from a large pediatric cohort.
• Sensory NCS data show no remarkable secular trend.

## 1 Introduction

Nerve conduction studies (NCS) are of integral importance in the differential diagnosis of childhood neuropathies (Eggermann et al., 2018, Rajabally and Nicolas, 2011). However, electroneurographical examinations in this patient population can be challenging.

In sensory NCS, a nerve containing sensitive nerve fibers is electrically stimulated at a position along its course. This leads to a depolarization of nerve fibers and can trigger an action potential. The evoked sum response oft the excited nerve fibers is then derived somewhere in the course of the nerve. For an artefact free recording an adequate muscular relaxation during NCS assessment is required (Wilbourn, 1994). The stimulus delivery can be uncomfortable and, therefore, compliance is often not given when children are examined. In case of an urgent indication for a NCS diagnostic, analgosedation can be considered (Ryan et al., 2019, Spires et al., 2017). Propofol and midazolam are among the most used sedativa for a paediatric population (Alletag et al., 2012, Krauss et al., 2014, Sauer et al., 2016, Sauer et al., 2020). However, these substances may influence sensory NCS parameters. In animal studies the excitability of primary sensory afferents was significantly reduced by Propofol and Midazolam in-vitro. Recordings of the sciatic nerve of rats showed a significant decrease of the area under the curve (AUC) of the stimulus response potential by midazolam (Neukom et al., 2012, Yilmaz et al., 2014, Yilmaz et al., 2000, Yilmaz-Rastoder et al., 2012). One human study of the median nerve using threshold tracking showed nonspecific alterations of some excitability parameters by propofol, mainly caused by technical issues (Maurer et al., 2010). To our knowledge, more human sensory NCS studies on the effect of systemic administration of sedativa are lacking.

Also large group studies of NCS in healthy pediatric populations are scarce and were mainly performed in the 1990s (Cai and Zhang, 1997, Garcia et al., 2000, Hyllienmark, 1995, Parano et al., 1993, Rabben, 1995). Anthropometric variables, along with the age, are important factors influencing NCS parameters during the growth and maturation phases (Hyllienmark, 1995). Particularly, in some premature age ranges, height and body weight have shown a marked acceleration trend in the industrial countries during the 20th century. Although this trend showed a stagnation in terms of height, it continued in terms of body weight (Cole, 2000, Gohlke and Woelfle, 2009, Hesse et al., 2016, Schönbeck et al., 2013, Zellner et al., 2004). This raises the question whether NCS normative data collected in the 1990s can be projected to the contemporary child and adolescent population.

In the present study, we investigated the influence of sedativa on sensory NCS parameters. For clinical use, we propose updated normative data of sensory NCS for a pediatric population.

## 2 Methods

### 2.1 Study population

In a prospective cross-sectional study from 2017 to 2019, 182 children (100 male and 82 female, aged 12 months to 18 years) were examined. The study was approved by the local ethics committee of the medical faculty of Otto von Guericke University Magdeburg (registration number 39/17) and written consent was received by the parents.

During the study period all children underwent elective surgery and received general anaesthesia in the Department of Otorhinolaryngology or in the Department of Pediatric Surgery at the University Clinic, Otto-von-Guericke-University Magdeburg. The most common surgical indications were adenotomies (n = 78) and removal of osteosynthetic material (n = 31). NCS measurements were performed under general anaesthesia (total intravenous (n = 151), combined volatile-intravenous (n = 31), muscle relaxation for intubation (n = 88)). The examination was performed unilaterally (right or left) at an accessible extremity not interfering the surgical procedure or other processes in the operating room.

Children with known hereditary peripheral or central nervous system disorders, abnormalities in motor development, trauma of the examined extremity, nutritional deficiencies, diabetes mellitus, and the presence of other chronic diseases were excluded from the study.

### 2.2 Nerve conduction studies

Sensory NCS of the median and sural nerves were recorded in 182 children during general anaesthesia and in 47 of these without anaesthesia post-surgery. To provide replicable conditions, the studies were always performed in the supine position with full extension of the extremities.

**Fig. 1:**
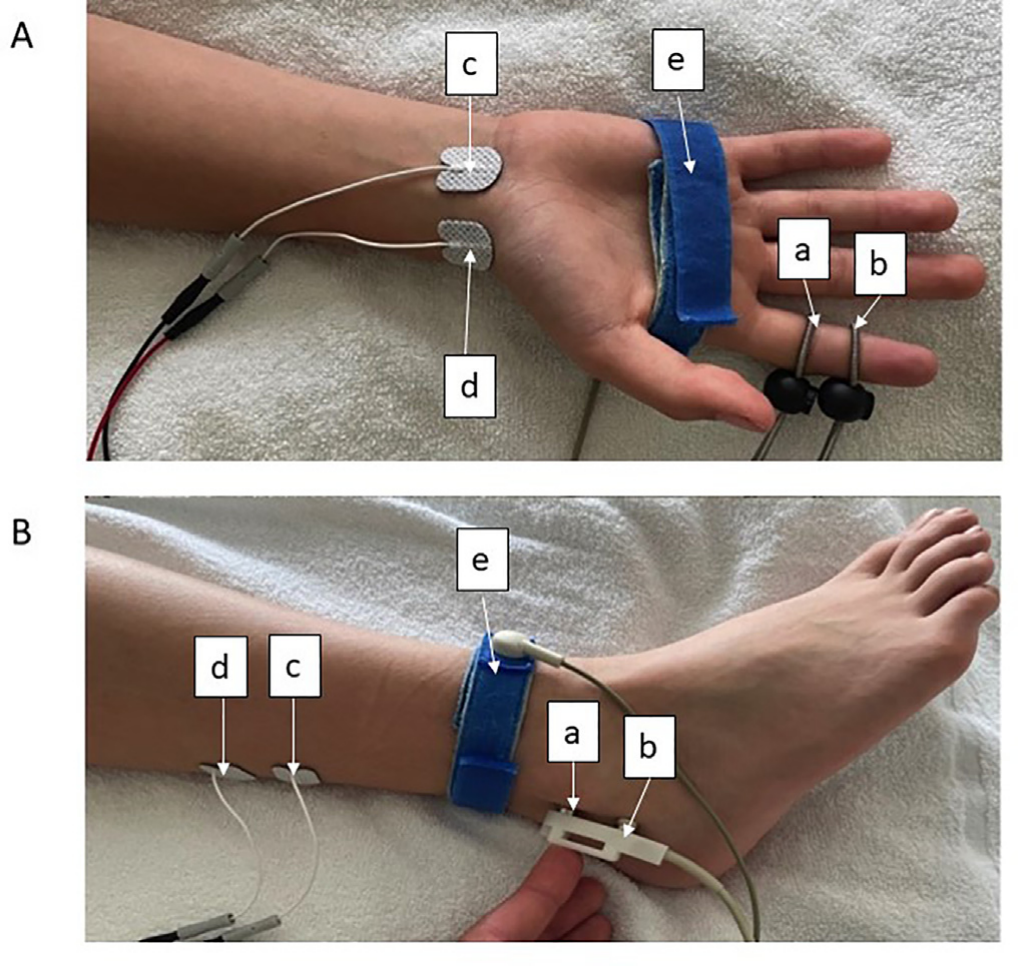
Electrode positions for orthodromic sensory NCS of the median nerve using a ring electrode (A) and sural nerve using a block electrode (B). a: cathode (stimulation), b: anode (stimulation), c: cathode (registration), d: anode (registration), e: ground electrode.

Technical setting included a Medtronic Keypoint G3 (software Keypoint.Net 5.11), for stimulation a bipolar surface block electrode (inter electrode distance 23 mm) for sural nerve respectively a bipolar ring electrode for median nerve and for recording pre-galled Ag/AgCl patch-electrodes (recording area 263 mm^2^) at standardized electrode positions (Table 1***,*** Fig. 1).

**Table 1:** Electrode positions of sensory NCS of median and sural nerve. * The measuring section was determined between the center of the stimulation anode and the center of the differential recording electrode. Abbreviation: NCS: nerve conduction study, SD: standard deviation.

	**Median Nerve**	**Sural Nerve**
stimulation	ring electrodes over index finger	block electrode between lateral malleolus and hamstring
recording	patch electrode at wrist between flexor carpi ulnaris and palmaris muscle tendons	patch electrode distal of gastrocnemius muscle and 1–2 cm lateral of midline
Age dependend measuring section* mean (SD)/ mm	1–2 years: 89 (7) 2–4 years: 96 (7) 4–6 years: 104 (7) 6–14 years: 126 (16) 14–18 years: 148 (10)	1–2 years: 75 (15) 2–4 years: 75 (14) 4–6 years: 93 (15) 6–14 years: 100 (19) 14–18 years: 106 (21)
reference	patch electrode at wrist medial to ulnar styloid processus	patch electrode 2–3 cm proximal of recording electrode
grounding	between stimulation and recording electrode around the center of the palm	between stimulation and recording electrode

**Fig. 2:**
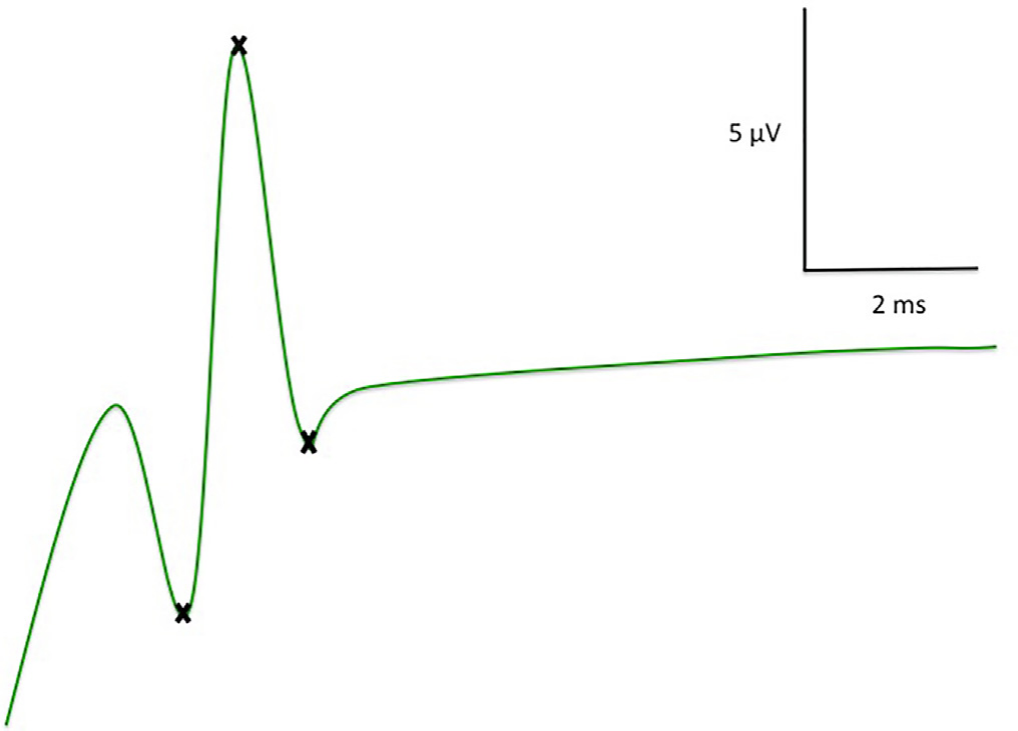
Sural nerve fiber sum action potential examined in an 8-year-old boy (amplitude: 11 µV, conduction velocity: 50 m/s).

An orthodromic examination technique was used and supramaximal stimulation was applied with pulses of 0.1–0.2 ms duration and 1 Hz frequency. At least 30 individual responses were averaged two times (frequency filter: 20 Hz to 2 kHz, display sensitivity: 5 µV/ 0.7 cm and 2 ms/ 0.7 cm). Latencies were calculated from the stimulus onset to the first positive peak for the determination of nerve conduction velocity (NCV), and amplitudes (SNAP) were measured peak-to-peak (Fig. 2). During the course of the study, we observed that children did not tolerate a supramaximal stimulation for NCS measurement without anaesthesia. Hence, the study protocol was adjusted. Additionally, in 31 of the 47 cases a submaximal stimulation during anaesthesia as well as in a waking state was applied (stimulus currents difference between both conditions < 0.5 mA).

Secondary exclusion criteria during the study performance included a surface temperature < 30 °C of the examined extremity, stimulation artifacts, a positive prewave of the measured response potential, and coexcitation of adjacent nerves.

### 2.3 Statistical analyses

Data was analyzed using SPSS version 25 and SAS University version statistical software. Effects were identified as non-significant if p ≥ 0.05.

The Wilcoxon test for connected samples with test value T was used for comparison of NCS results during and without anaesthesia.

For comparison with the study results of the 1990 s the sample was divided by age into five groups (1–2 years, 2–4 years, 4–6 years, 6–14 years, 14–18 years).

The age-dependent regression model with the smallest sum of squares of variance as well as the highest coefficient of determination was estimated for each NCS parameter. Linear, quadratic and cubic models were considered when the estimation and their regression constants in the ANOVA test were statistically significant. In addition, it was tested whether the corrected coefficient of determination could be improved by adding height and gender as independent variables. The reference range of the NCV parameter was defined by the 95 % prediction interval of the regression model.

## 3 Results

**Fig. 3:**
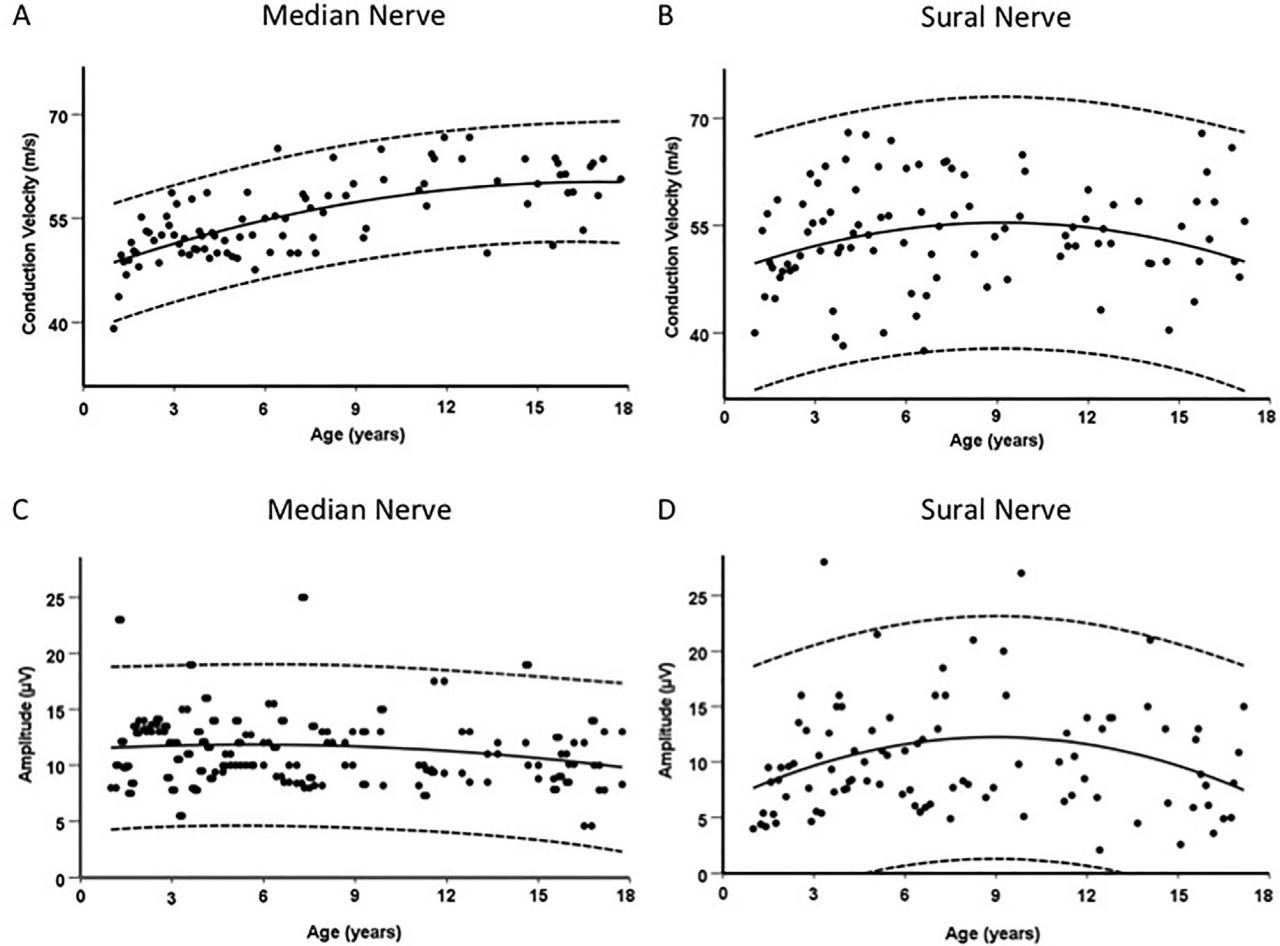
Nerve conduction velocity of median nerve (A) and sural nerve (B) and amplitude of median nerve (C) and sural nerve (D) in age dependent scatter charts. The solid lines show the corresponding regression model, the dashed lines show the associated 95% confidence interval.

After selection, 148 (of 150) examinations of the median nerve and 160 (of 173) examinations of the sural nerve were included in statistical analyses. The correlation between age and height was strong (R = 0.96). Adding height and gender as independent variables to the age did not influence the corrigated coefficient of determination of the following regression models (Fig. 3):

- median nerve NCV = 47.13 m/s + 0.130 x age − 3.15 x10^-4^ x age^2^ (corrected R^2^ = 0.43),
- sural nerve NCV = 44.00 m/s + 0.338 x age − 0.003 x age^2^ + 7.817 x10^-6^ x age^3^ (corrected R^2^ = 0.03),
- median nerve SNAP = 11.411 µV + 0.13 x age − 9.434 x10^-5^ x age^2^ (corrected R^2^ = 0.01),
- sural nerve SNAP = 6.443 µV + 0.107 x age + 4.95x10^-4^ x age^2^ (corrected R^2^ = 0.05).

Only for the sensory median nerve NCV there is a significant age dependence (corrected R^2^ = 0.43) in our collective. Sensory NCV already reaches 86 % (sural nerve) respectively 82 % (median nerve) of the maximum of all age groups in age group 1–2 years. The maximal mean values of the sensory NCV are reached at age 4–6 years for the sural nerve and at age 14–18 years for the median nerve. Average SNAP of sural nerve showed an age dependent increase from age group 1–2 to 2–4 years, no relevant changes were found for the median nerve (Fig. 3***,*** Table 2***,*** Table 3).

**Table 2:** Age dependent values of sensory nerve conduction velocity. Comparison of present results with former studies (All studies listed determined the latency to the first negativ deflection of the stimulus response).

Nerve/ age group	Present results	Parano et al., 1993	Garcia et al., 2000	Ryan et al., 2019	Martinez et al., 1978
	Sensory Nerve Conduction Velocity (m/s)mean (SD)				
Median Nerve					
1–2 y	48.7 (3.8)	46.9 (5.0)	45.1 (3.0)	55 (6)	59.7 (6.13)
2–4 y	52.4 (3.4)	49.5 (3.3)	48.8 (3.0)	59 (6) (2-3y)	66.4 (5.0)
4–6 y	52.2 (4.,4)	51.7 (5.2)	50.7 (3.6)	61(5) (3-5y)	63.4 (4.18)
6–14 y	573 (5.5)	53.8 (3.3)	n.a.	64 (5) (5-10y)	66.2 (3.89)
14–18 y	59.7 (4.5)	n.a.	n.a.	65 (4) (15-18y)	n.a.
Sural Nerve					
1–2 y	49.1 (8.9)	49.7 (5.5)	n.a.	n.a.	n.a.
2–4 y	51.6 (9.7)	52.6 (3.0)	n.a.	57 (5) (2-5y)	n.a.
4–6 y	57.3 (8.9)	53.8 (4.3)	n.a.	n.a.	n.a.
6–14 y	54.2 (7.4)	53.9 (4.2)	n.a.	56 (7) (5-10y)	n.a.
14–18 y	51.8 (8.7)	n.a.	n.a.	51 (5) (15-18y)	n.a.

Abbreviations: n.a.: not available, SD: standard deviation, y: years.

**Table 3:** Age dependent values of sensory nerve action potential (SNAP) amplitude. Comparison of present results with former studies. (Most studies determined the SNAP amplitude from the maximal positive peak to the maximal negative peak. *Parano et al. determined SNAP amplitude from baseline to peak.).

Nerve/age group (Years)	Present results	Parano et al., 1993	Garcia et al., 2000	Ryan et al. 2019	Chai and Zhang 1997
	Amplitude of Sensory Nerve Action Potential (µV)mean (SD)				
Median Nerve					
1–2 y	11,0.3 (4.4)	24.0 (7.4)*	15.7 (4.5)	54 (23)	19.5 (4.0)
2–4 y	11.9 (3.5)	24.3 (5.5)*	12.2 (5.9)	62 (24) (2-3y)	
4–6 y	11.5 (3.2)	25.1 (5.2)*	14.4 (6.0)	54 (20) (3-5y)	19.8 (4.2)
6–14 y	11.8 (3.6)	26.7 (9.4)*	n.a.	55 (19) (5-10y)	20.5 (3.5)
14–18 y	10.4 (3.5)	n.a.	n.a.	56 (18) (15-18y)	n.a.
Sural Nerve					
1–2 y	7.3 (3.9)	15.4 (8.2)*	n.a.	20 (7)	18.0 (3.8)
2–4 y	10.7 (5.7)	23.3 (6.8)*	n.a.	21 (9) (2-5y)	
4–6 y	11.1 (6.4)	22.7 (5.4)*	n.a.		18.5 (3.9)
6–14 y	11.0 (5.9)	26.8 (6.6)*	n.a.	21 (10) (5-10y)	18.7 (4.4)
14–18 y	9.7 (4.8)	n.a.	n.a.	21 (9) (15-18y)	n.a.

Abbreviations: n.a.: not available, SD: standard deviation, y: years.

Comparing sensory NCS with and without anaesthesia, no significant differences were observed. NCV and SNAP showed slightly but not significantly higher mean values during anaesthesia, most pronounced for the SNAP with about 15 % higher amplitudes during anaesthesia (Table 4).

**Table 4:** Comparison of NCS parameters with and without anaesthesia by Wilcoxon-statistics. Abbreviations: NCV: nerve conduction velocity; SD: Standard deviation, SNAP: sensory nerve action potential.

	**Base-NCV** (m/s)	**Peak-NCV** (m/s)	**SNAP-duration** (ms)	**Base-to-Peak-SNAP (**µV)	**Peak-to-Peak-SNAP (**µV)
	Mean (SD)	Mean (SD)	Mean (SD)	Mean (SD)	Mean (SD)
Anaesthesia (N = 43)	53.9 (4.5)	43.1 (4.9)	1.2 (0.16)	7.0 (3.3)	7.1 (3.4)
Awake (N = 43)	53.2 (4.2)	42.8 (4.9)	1.1 (0.14)	6.0 (2.7)	6.2 (2.7)
p (Wilcoxon)	0.40	0.15	0.07	0.13	0.19

## 4 Discussion

The comparison of sensory NCS data with and without anaesthesia revealed no influence of midazolam and propofol on sensory stimulus response. In contrast, in vitro animal experiments showed a reduction of the A-fiber excitability under the influence of these substances (Neukom et al., 2012, Yilmaz et al., 2014, Yilmaz-Rastoder et al., 2012) which can lead to a reduction of NCV and SNAP (Beyazova et al., 2011, Neukom et al., 2012, Pereon et al., 1999). These animal studies were performed on isolated nerves in-vitro and the threshold tracking method was used. This study design is more sensitive to detect nerve excitability changes than conventional NCS used here, therefore a direct comparison between both methods is not possible.

Indeed, the mean values of SNAP amplitude during anaesthesia were higher, which could be related to optimized measurement conditions during anaesthesia. Multiple measurements were possible without any cooperation of the tested child.

The interpretation of these data has two limitations: First the transfer into diagnostic anxiolysis or sedation with benzodiazepines is limited, because compared to other studies (Maurer et al., 2010, Neukom et al., 2012, Wang et al., 2020), this cohort received midazolam in lower doses through longer time intervals. Second, the reproducibility could be limited because submaximal stimulation causes a higher variance of the NCV as well as of the SNAP (Nashed et al., 2009). However, it was tried to minimize this effect by using nearly equal stimulation current levels, both awake and under anaesthesia.

Like in other studies an age dependent increase of NCV up to age 6–14 years (median nerve) and 4–6 years (sural nerve) was observed (Garcia et al., 2000, Parano et al., 1993). The SNAP values reached approximately maximum for age 1–2 years (median nerve) and for age 2–4 years (sural nerve), respectively (Cai and Zhang, 1997, Garcia et al., 2000, Parano et al., 1993) (Table 3). For the age 14–18 years no data are available from the former studies (Garcia et al., 2000, Parano et al., 1993).

A comparison with normative data from the 1990s is limited, because all subjects underwent elective surgery and the present cohort is not entirely representative of the general healthy population.

In our data the sensory median nerve NCV were higher - in some cases more than one standard deviation - then in pediatric populations from the 1990s (Garcia et al., 2000, Parano et al., 1993, Rabben, 1995) matching a retrospective analysis with a recent data collection from 1997 to 2017 (Ryan et al., 2019). Results from earlier studies are inhomogeneous. In line with our trend, median nerve NCV were significantly lower in one earlier study (Gamstorp and Shelburne Jr, 1965), but in contrast, another earlier study obtained higher NCV values (Martinez et al., 1978). This variability of median nerve NCV between the different studies cannot be explained conclusively. Longer latencies with an antidromic regristration technique should be taken into account (Bolton and Carter, 1980, Chouhan et al., 2021, Murai and Sanderson, 1975, Valls-Sole et al., 2016, Cohn et al., 1990), but most studies used the orthodromic technique (Gamstorp and Shelburne, 1965, Garcia et al., 2000, Parano et al., 1993, Rabben, 1995) and only two studies examined with the antidromic technique (Martinez et al., 1978, Ryan et al., 2019). Furthermore all studies investigated nerve segments between wrist and digit II and III respectively and controlled skin temperatures to be higher than 30.5 °C (Garcia et al., 2000, Martinez et al., 1978, Parano et al., 1993, Ryan et al., 2019).

The higher variance of the sural nerve NCV compared with previous studies (Garcia et al., 2000, Parano et al., 1993) can be attributed to different registration techniques. Parano et al. used smaller electrodes for children and newborns compared to our setting (Parano et al., 1993). Larger electrode areas used here may amplify the effect of volume conduction which makes the determination of the first deflection of the stimulus response difficult. In this cohort 7 of the 173 investigations had to be excluded due to stimulation artifact or positive prewave of the measured response potential.

The mean values of the SNAP amplitude of our collective are up to two standard deviations lower than in former studies (Parano et al., 1993, Cai and Zhang, 1997, Ryan et al., 2019, Garcia et al., 2000). Using antidromic and/ or bipolar registration could explain the higher SNAP amplitudes in those studies (Parano et al., 1993, Cai and Zhang, 1997, Ryan et al., 2019). Yet we have no technical explanation for the higher SNAP amplitudes of a study also using a monopolar and orthodromic registration technique (Garcia et al., 2000).

It is conceivable, that a secular trend towards increasing body weight is involved in the trend towards lower amplitude values in our collective (Bolton and Carter, 1980, Schulte-Mattler et al., 2001, Stetson et al., 1992), because a negative correlation of body weight or finger circumference with median nerve SNAP has been described (Bolton and Carter, 1980, Hasanzadeh et al., 2008, Stetson et al., 1992). Incongruously, there is no clear correlation of NCV with body size independent of age or body weight, so that the higher NCV values in the present data cannot be plausibly explained by a secular trend of anthropometric data (Hyllienmark, 1995, Lang et al., 1985, Buschenbacher, 1998).

Taken together, NCS differences of studies cannot be attributed solely to a secular trend of anthropometric data, they are also caused by methodological differences.

## 5 Conclusion

Despite their limitations the present data suggest - as shown for motor NCS (Dueck et al., 2003, Kaieda et al., 1981, Kerz et al., 2001) - that sedation could be used to improve the sensory NCS in clinical practice. Conventional sensory NCS parameters are not significantly affected by benzodiazepine and propofol sedation contrary to more sensitive methods like threshold tracking and in-vitro measurement.

Differences of the present data in comparison with former studies are mainly attributed to technical differences. In some extend the influence of a secular trend of anthropometric data cannot be excluded. However, due to the different measurement techniques, a clear trend is uncertain.

The here provided sensory NCS data from a large pediatric cohort with standardized measurement technique facilitate the use in clinical practice.

## Declaration of competing interest

The authors declare that they have no known competing financial interests or personal relationships that could have appeared to influence the work reported in this paper.
